# How repeated and unpredictable experiences turn into fear-memories

**DOI:** 10.3389/fnins.2013.00017

**Published:** 2013-02-21

**Authors:** J. M. Pêgo, N. Sousa

**Affiliations:** ^1^Life and Health Sciences Research Institute (ICVS), School of Health Sciences, University of Minho, Campus de GualtarBraga, Portugal; ^2^ICVS/3B's - PT Government Associate LaboratoryBraga/Guimarães, Portugal

The role of the amygdala in regulating emotional neural processing has been well-acknowledged by both animal and clinical studies (LeDoux, 2007; Sehlmeyer et al., 2009; Morrison and Salzman, 2010), particularly those relating to aversive stimuli that characterize fear behavior. Anxiety and fear disorders (American Psychiatric Association, 2000) constitute a subset of mental disorders characterized by feelings of uneasiness and apprehension, rumination, and excessive worrying about real or imaginary threats. The clinical spectrum ranges from generalized anxiety disorder to specific phobias. While sharing many behavioral features these disorders are distinguishable by the specificity of the cues that elicit symptoms (American Psychiatric Association, 2000). Much of the knowledge that we have about fear and anxiety stems from several animal models using fear-conditioning paradigms (Shekhar et al., 2001; Mechiel Korte and De Boer, 2003). Typically an unconditioned and clearly aversive stimulus (e.g., footshock) is associated to a conditioned neutral stimulus (e.g., light or sound) until an association is established (Johansen et al., 2011). Later, the memory of that fearful event is tested by presenting the conditioned stimulus and observing similar behavioral responses to those presented during the learning phase. While these models are useful and robustly reproducible they fall short of resemblance to the human expression of anxiety disorders. In fact, most patients fail to establish a direct association between a specific cue or moment and the development of symptoms (American Psychiatric Association, 2000).

In an interesting article “*The role of the lateral amygdala in the retrieval and maintenance of fear-memories formed by probabilistic reinforcement*” published in Frontiers in Behavioral Neuroscience, Erlich and colleagues (2012) provide the first evidence for the role of the amygdala in the establishment and maintenance of fearful memories in response to aversive stimuli that are presented in a probabilistic pattern instead of the classical pavlovian deterministic way. Specifically, these authors used a partial reinforcement paradigm where a conditioned stimulus probabilistically predicts the unconditioned stimulus in rats' that where pharmacologically manipulated to alter the activity of the lateral nucleus of the amygdala and reported very interesting results. First, using the lick suppression ratio in response to CS the authors established a model in which both increases and decreases in fear response could be assessed. Secondly, by surgically implanting cannulas in the lateral nucleus of the amygdala they were able to alter fear-conditioning expression using muscimol, a GABA-A receptor agonist, and pentagastrin, a CCK2 receptor antagonist. While muscimol was able to almost completely abolish the lick suppression ratio induced by the CS, pentagastrin did not alter this behavior. This result is in accordance with previous reports using classical fear-conditioning models (Johansen et al., 2011) and clinical evidence from the use of therapeutic agents acting on GABA receptors like benzodiazepines (Reinhold et al., 2011). However, stimulation of lateral amygdala by pentagastrin induced an “all or nothing” effect, where some trials induced a “high fear” expression and others had no effect at all; importantly, in the trials that showed a positive effect, a long-lasting suppression of licking was observed.

The main outcome of this study is the demonstration that lateral amygdala activity is essential to the expression of fear-behavior in probabilistic paradigms and that CCK2 receptor activation may lead to impaired recovery from fearful memories. This is the first demonstration of this role and sheds light on new therapeutic targets in the modulation of the fear response in a post-encounter approach.

Yet, the most remarkable finding reported by Erlich and colleagues is the role of lateral amygdala in the encoding of uncertain information using a probabilistic presentation of the aversive stimulus. It has been well-characterized that the lateral amygdala has a paramount role and is the neuroanatomical substrate (Phelps and LeDoux, 2005; Johansen et al., 2011) for the encoding of the association of aversive-neutral stimuli that characterize fear paradigms. Although these approaches are useful for experimental purposes it is less clear if the outcomes stemming from such reports can be extrapolated to the clinical setting. The development of anxiety disorders is commonly indolent in its presentation and seldom is established following the presentation of overtly aversive stimuli (American Psychiatric Association, 2000). Importantly, the unpredictability of the aversive event may in fact result in stronger and longer lasting associations (Hubbard et al., 2011). Although it has been postulated that other brain regions are associated to anxiety disorders while amygdala functions were ascribed to fear behavior (Walker et al., 2003), this report clearly establishes a role for the amygdala in anxiety disorders. It is likely that it acts by establishing the emotional value of the stimulus while modulating downstream circuitries, namely the bed nucleus of the stria terminalis (Pêgo et al., 2006).

In conclusion, the findings reported by Erlich and colleagues represent an appealing topic of investigation from both behavioral and pharmaceutical points of view in that it provides a rationale of developing in the future chemical compounds that, by manipulating lateral amygdala function in particular through the modulation of CCK2 receptors, may ameliorate negative and aversive emotional states.
